# Characteristics and control measures of odor emissions from crematoriums in Beijing, China

**DOI:** 10.1007/s42452-021-04738-7

**Published:** 2021-07-26

**Authors:** Yangyang Cui, Xiaoman Zhai, Baocheng Wang, Shihao Zhang, Amanzheli Yeerken, Xizi Cao, Lianhong Zhong, Liming Wang, Tong Wei, Xinyu Liu, Yifeng Xue

**Affiliations:** 1 National Engineering Research Center of Urban Environmental Pollution Control, Beijing Municipal Research Institute of Environmental Protection, Beijing, China; 2 Key Laboratory of Pollution Control of Ministry of Civil Affairs, 101 Institute of Ministry of Civil Affairs, Beijing, China; 3 Beijing Municipal Solid Waste and Chemical Management Center, Beijing, China; 4 3Clear Science & Technology Co., Ltd, Beijing, China; 5 Babaoshan Funeral Parlor, Beijing, China

**Keywords:** Crematorium, Odor concentration, Emission level, Influencing factors, Control countermeasures

## Abstract

The promulgation and implementation of the national and Beijing municipal standards for air pollutants emitted from crematoriums has effectively alleviated the problem of “black smoke” in crematoriums, but noticeable odor in crematoriums remains. We determined the level of odor emissions in crematoriums by monitoring the odor concentrations of cremators, incinerators, and cremation workshops in five crematoriums in Beijing. Subsequently, we analyzed the major contributing factors to the odor level and proposed control measures. A high odor concentration in crematoriums was observed; two different mechanisms were proposed to explain this finding. First, poor ventilation conditions in workshops and inadequate airtightness of equipment resulted in dimensionless concentrations of unorganized odor emissions in the workshops ranging from 97 to 732, with an average of 504, which is much higher than the standard level of 20. Second, the postprocessing facilities used in cremation sites produce poor odor removal, which, coupled with fuel usage and unregulated operations, led to high concentrations of organized odor emissions ranging from 231 to 1303 (910 on average) for cremators and incinerators. The odor emissions of cremators and incinerators meet the Integrated Emission Standards of Air Pollutants (DB11-501-2017), which are suitable for industries containing industrial kilns but not for crematoriums. The odor emissions in crematoriums are lower than those emitted from industries, such as fiber manufacturing and activated carbon processing. However, the unique geographical locations of crematoriums, high population density, and high exposure risk to local residents necessitate strengthening the management and control of odor emissions from crematoriums. To further address the problem of odor emissions from crematoriums in Beijing, further clarification and tightening of industry standards for the concentration limits of organized and unorganized odor emissions is recommended. Crematoriums will thus be prompted to increase odor control in workshops and adopt and improve deodorization facilities, including the installation and application of treatment facilities, such as adsorption and biological control.

## Introduction

Recent years have witnessed continuous growth in the number of cremations in China. This number reached 5.017 million by the end of 2018, representing a 9.3% increase from 2014, and continues to increase [1]. A large number of corpses and sacrificial items are incinerated in crematoriums, which have thus become a considerable source of air pollution. The cremation and incineration processes in crematoriums produce a large quantity of atmospheric pollutants, including particulate matter, carbon monoxide (CO), nitrogen oxides (NOx), sulfur dioxide (SO_2_), hydrochloric acid (HCl), volatile organic compounds (VOCs) and dioxins [2–4]. These air pollutants spread near the ground and impact the surrounding environment and human health. Following the promulgation and implementation of the Emission Standard of Air Pollutants for Crematoriums (GB 13,801-2015) and the Beijing Municipal Emission Standard of Air Pollutants for Crematoriums (DB11/1203-2015) in 2015, crematoriums effectively decreased the emissions of particulate matter and some gaseous pollutants through the upgrade and transformation of cremators and incinerators, as well as the installation of flue gas purification devices. These modifications have alleviated smoke and dust emissions from cremators and incinerators, i.e., “black smoke”, to some extent. However, the results of a field survey on crematoriums in Beijing show that odor remains rather strong. Thus, it is necessary to characterize and quantify the level of odor in crematoriums.

Previous studies have mainly focused on evaluating the emission characteristics and effectiveness of controls for particulate matter and gaseous pollutants emitted by crematoriums, including SO_2_, NOx, CO, and VOCs. Xue et al. used monitoring data for nine typical crematoriums in Beijing to determine the emission characteristics of smoke from crematoriums and emission factors and levels of various harmful air pollutants [5]. Zhang et al. investigated the emission characteristics of harmful substances by determining the quantity and chemical components of particulate matter, as well as the emission levels of gaseous pollutants, emitted from the incineration of sacrificial items [6]. Laura et al. used a case study to evaluate pollution from hazardous substances produced in crematoriums and human health risks based on air diffusion and health risk assessment models [7]. Cardoso et al. applied the isokinetic sampling method of the US Environmental Protection Agency 201A, to samples collected from crematoriums in Mexico and investigated the PM2.5 emission characteristics [8]. Takeda et al. analyzed changes in the emission factors in Japan from 1999 to 2007 for polychlorinated dibenzo-p-dioxins and polychlorinated dibenzofurans (PCDD/Fs) from crematoriums [9]. Thus, considerable attention has been paid to emissions of particulate matter, VOCs, NOx, and PCDD/Fs from crematoriums but studies have not been performed to determine the concentration and influencing factors for odors from crematoriums.

In this study, we investigated emission levels of odor from crematoriums by conducting onsite monitoring in five typical crematoriums in Beijing. We investigated organized emissions of cremators and incinerator chimneys, as well as unorganized emissions from cremation workshops. We used laboratory analyses to produce monitoring data to determine the emission levels and influencing factors. Based on these results, we identified the industry emission reduction potential and proposed control countermeasures for improving industry standards.

## Materials and methods

### 1.1 Research subject and monitoring points

**Fig. 1:**
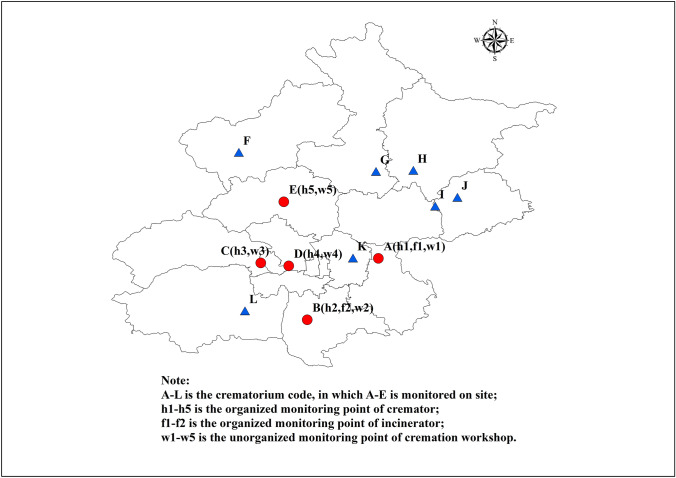
The spatial distribution of Crematoriums in Beijing

There are 12 crematoriums in Beijing that cremate 90,000–100,000 corpses annually. The spatial distribution of crematoriums is shown in Fig. 1. The monitoring points were selected based on the requirements for the determination of particulate matter in the exhaust of fixed pollution sources and the sampling method for gaseous pollutants (GB/T 16157). The sampling locations of the organized emissions were set at the straight sections of chimneys to avoid elbows and vortex areas and thereby ensure uniform gas collection. The sampling locations of the unorganized emissions were set in the operation areas of the cremation workshops. The cremators were operated for a period of time before sample collection to ensure that the odor level of the samples were largely consistent with that affecting the health of the operators. Basic information for the five crematoriums monitored in this study is shown in Table 1. The main flue gas purification process was cooling, acid removal through spraying, and dust removal (using a cyclone and a cloth bag). Five samples were obtained from cremators, five samples were obtained from cremation workshops, and two samples were obtained from incinerators.

**Table 1:** Basic information of crematoriums in Beijing

Crematorium	Monitoring points of organized discharge		Monitoring points of disorganized discharge	Post-processing
	Cremation	Incinerator	Cremation workshop	
A	h_1_	f_1_	w_1_	Air cooling + Cyclone dust removal + Bag-type strainer + Air fan
B	h_2_	f_2_	w_2_	Water cooling + Cyclone dust removal + Bag-type strainer
C	h_3_	/	w_3_	Cooling + Spray acid + Bag-type strainer
D	h_4_	/	w_4_	Cooling + Heat transfer + Spray acid + Bag-type strainer
E	h_5_	/	w_5_	Cyclone dust removal + Bag-type strainer + Fume hood + Exhaust

### Mechanism of odor generation in crematoriums

**Fig. 2:**
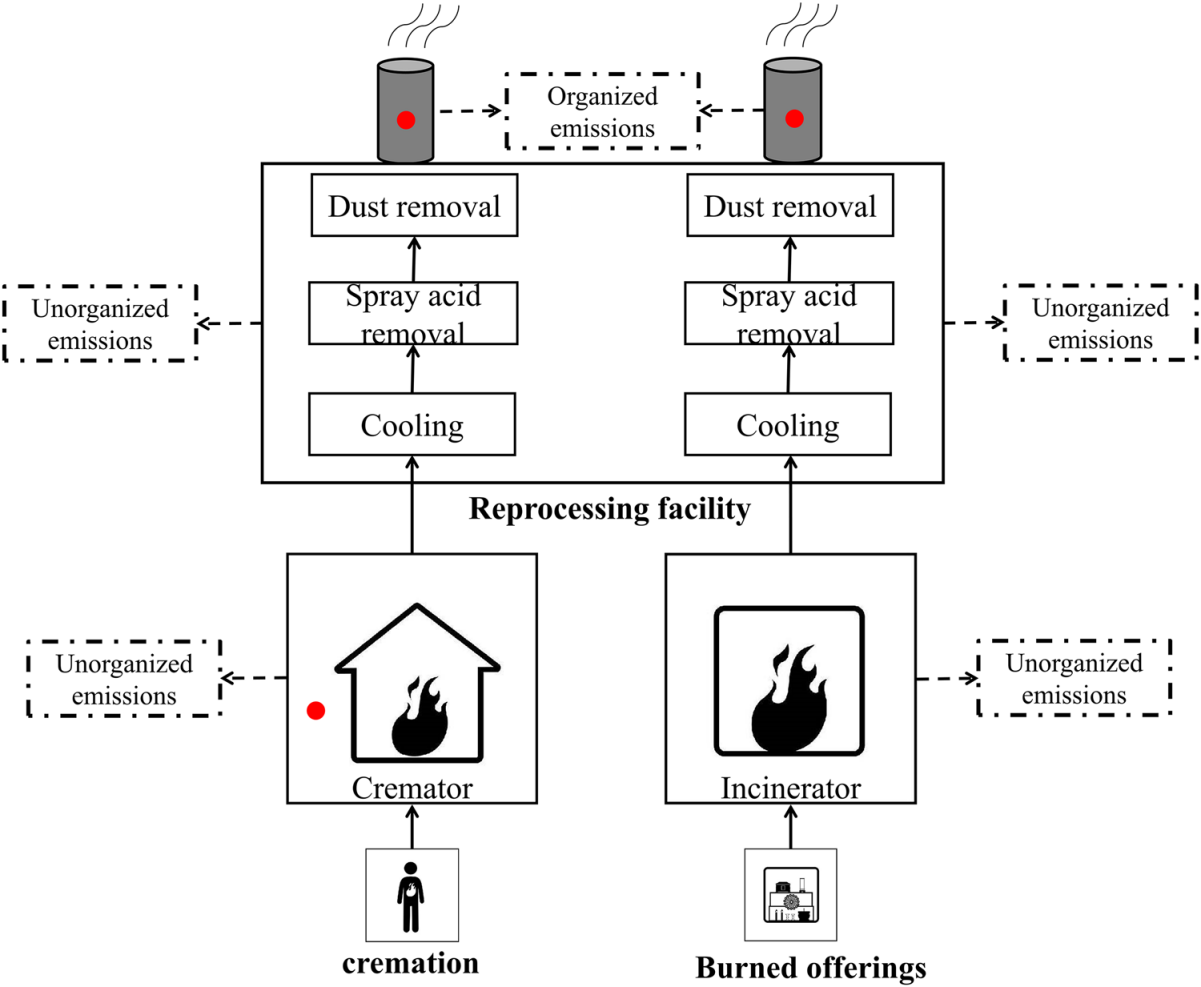
The principle of crematorium odor production

Incomplete combustion during the cremation of human corpses and the incineration of relicts and sacrifices produce odor emissions, which usually include odorous gases, such as ammonia (NH_3_), hydrogen sulfide (H_2_S), styrene, carbon disulfide, and methyl mercaptan. Human corpses disintegrate under heat within the first 5 min of being placed in a cremator, and gas production peaks 10 min after the body enters the cremator, emitting the highest volume of odorous gases. Industrial cremation is a non-steady state process with variable boundaries and intermittent combustion. Thus, the emission channels are scattered, with many sources of unorganized emissions, and the emitted gases are difficult to collect and easily diffuse into the environment [10–12]. Currently, crematoriums do not have specialized odor treatment equipment, and existing posttreatment facilities have poor odor removal capabilities. Thus, some odor is discharged to the atmosphere through chimneys in the form of organized emissions. In addition, incomplete collection produces some unorganized odor emissions in workshops and at the crematorium boundaries, which impacts the workshop staff, residents in surrounding areas, and environment. The mechanism of odor generation in crematoriums is shown in Fig. 2 (the study monitoring points are shown as red dots).

### Odor monitoring method

We used air bags to collect samples of odorous gases emitted from the chimneys of cremators, the chimneys of incinerators, and the cremation workshops of the five investigated crematoriums. To prevent the original air in the air bags from affecting the results, the air bags were flushed at least thrice with nitrogen before sampling, and the samples were collected using an air-bag vacuum sampling box (Model 1062) [13]. The odor concentration was analyzed using the odor-triangle odor bag method for the determination of air quality (GB/T 14675-1993). The method consists of diluting a sample stepwise. After each dilution, an air bag filled with the sample gas is handed to a sniffer along with two air bags filled with odorless air. The sniffer identifies which air bag is odorous. Then, a judge processes data for the results obtained for a group of sniffers (usually six people) to obtain the final sample odor concentration.

## Results and discussion

### The level and influencing factors of odor emissions from cremators

**Fig. 3:**
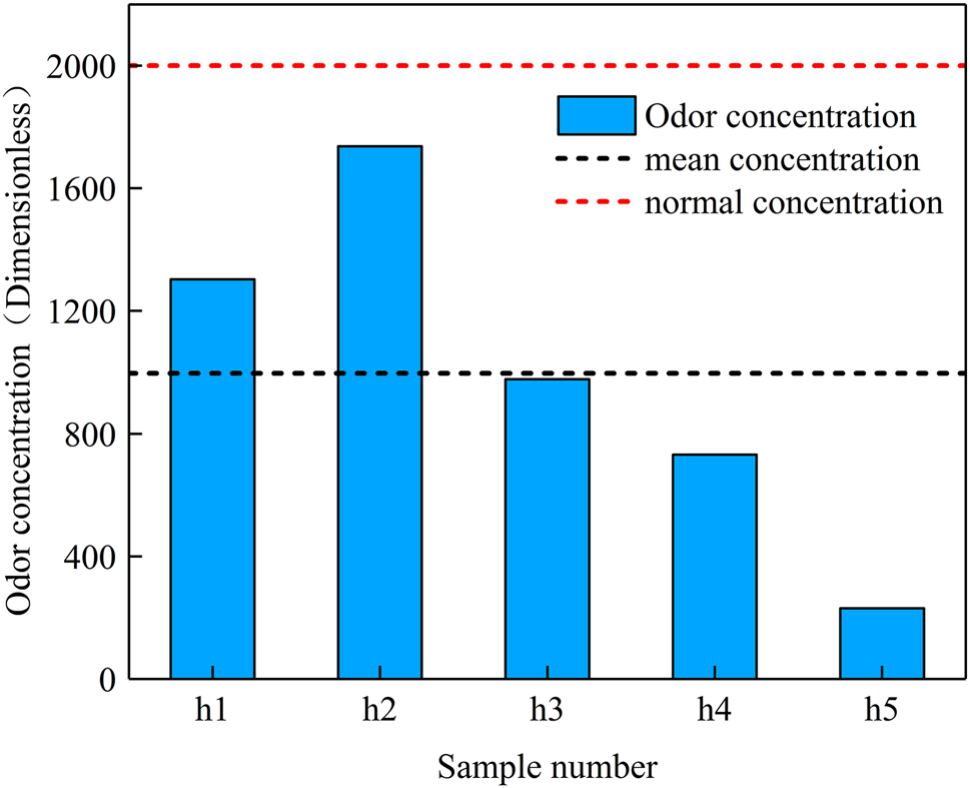
The level of odor emissions of cremators

After the release and implementation of the Beijing Municipal Emission Standard of Air Pollutants for Crematoriums (DB11/1203-2015) in 2015, most crematoriums in Beijing were upgraded and installed posttreatment facilities to control the emissions of gaseous pollutants from cremators to some extent. However, existing postprocessing facilities cannot adequately remove crematorium odor, resulting in highly concentrated organized odor emissions from cremators. The odor concentration range measured in this study was 231-1303 (996 on average) (Fig. 3). This result was obtained because the cremation temperature is generally insufficiently high and the exhaust gas retention time is insufficiently long. Improper operation and aging equipment commonly result in odor leakage and therefore a high odor concentration. At present, the cremators used in Beijing are still fuel-based and generate pungent odor during combustion, thus increasing the level of odor emissions. Most cremators in Beijing are equipped with a secondary combustion chamber that dilutes odor but not to a sufficient extent. Therefore, a clean-energy transformation of cremators needs to be implemented to decrease organized odor emissions, that is, fuel should be replaced with gas, and more purification facilities should be installed.

### The level and influencing factors of odor emissions from incinerators

**Fig. 4:**
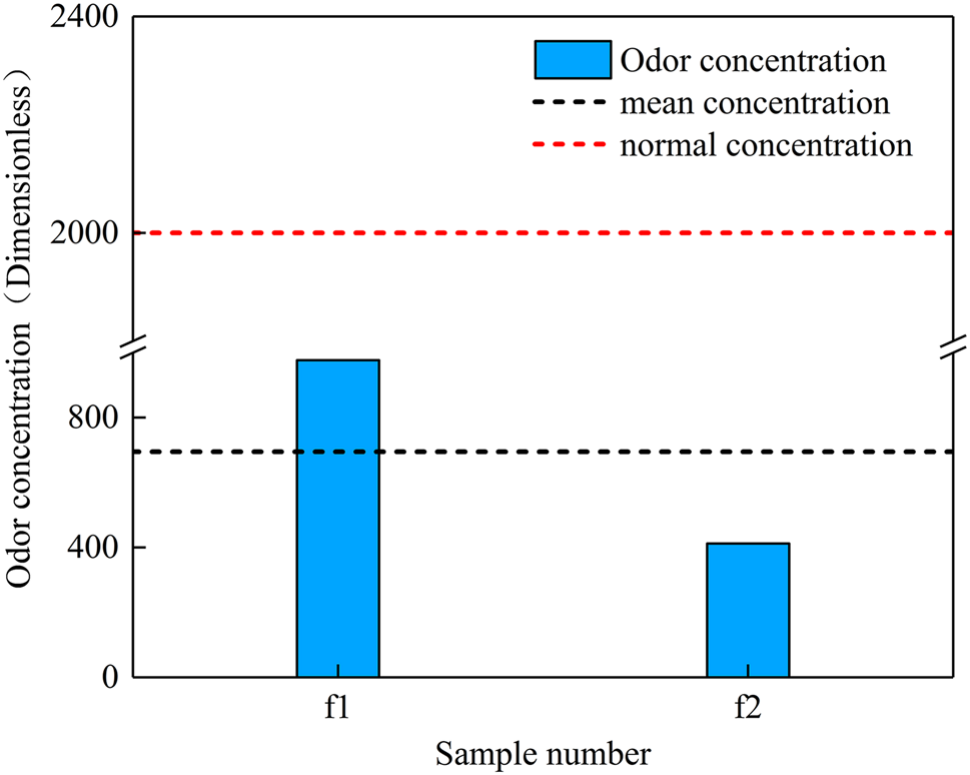
The level of odor emissions of incinerators

The emergence of COVID-19 has resulted in a ban on most incinerators. Thus, only two monitoring samples were obtained from incinerators in this study. Figure 4 shows the monitoring results of the odor concentration of the incinerators, which was in the range of 412-977 (695 on average). These values are lower than the current standard limit of the Integrated Emission Standards of Air Pollutants (DB11/501-2017) but corresponds to a higher emission level and human exposure risk than from other industries. Most of the 24 sacrificial incinerators in Beijing are out of service, because sacrificial incineration has been replaced by the placement of silk wreaths. Thus, a relatively limited quantity of monitoring data is available. The installation of purifiers in incinerators and the adoption of a variety of flue gas purifying measures have effectively controlled gaseous pollutant emissions, increasing the prominence of odor emissions. Disorganized management of sacrificial incineration in Beijing and the lack of auxiliary fuel in incinerators has increased odor production from the incomplete combustion of various sacrifices. As a result of inadequate operating and managing personnel, organized odor emissions from incinerators remains at a very high level. The implementation of policies on managing and controlling incinerator emissions has resulted in a considerable reduction in the number of sacrificial items over the past few years, but there is still room for improvement. Therefore, more deodorizing facilities for incinerators should be constructed, and personnel operations and equipment management should be standardized.

### The level and influencing factors of odor in cremation workshops

**Fig. 5:**
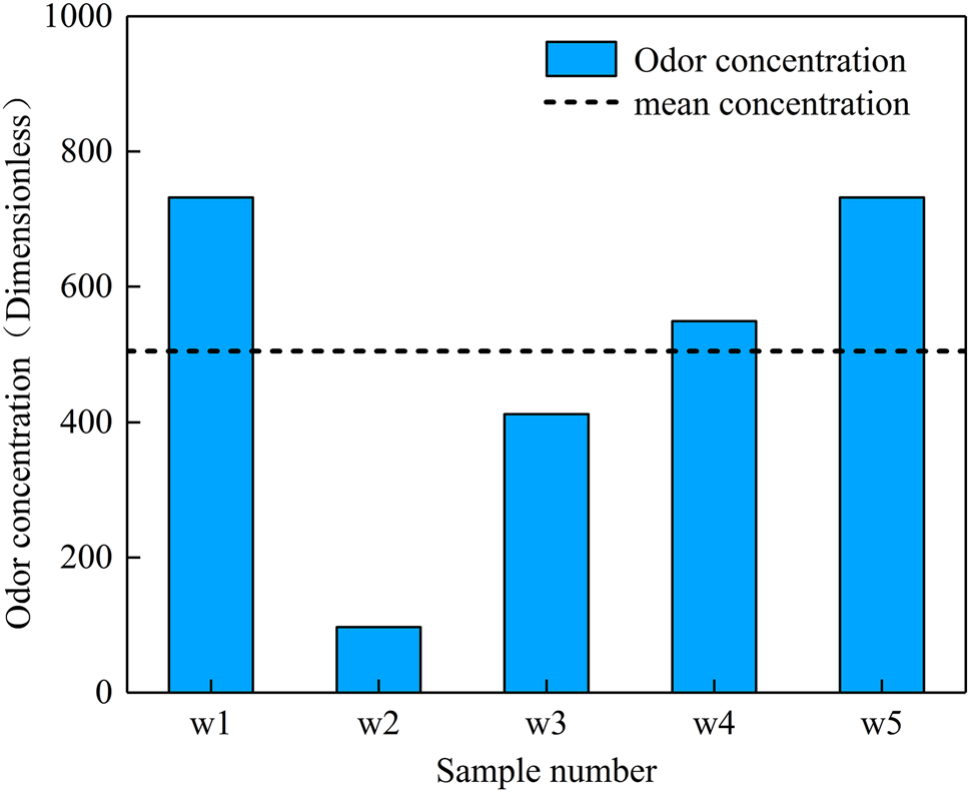
The odor level in cremation workshops

In Beijing, cremators, incinerators, and postprocessing devices are all installed in cremation workshops and operated indoors. Consequently, a large quantity of unorganized odor emissions accumulate inside the workshop and impact the health of the workshop staff. Data from various monitoring points in the cremation workshops of five funeral homes in Beijing are shown in Fig. 5. The unorganized odor concentration in the cremation workshops was 97-732 (504 on average), indicating a high emission level that should be attended to. This result was obtained because cremation workshops are typically closed spaces with poor ventilation. Thus, odor generated by the cremation process leaks into the workshop and is not promptly ventilated, resulting in a gradual increase in the level of unorganized odor emissions in the workshop. In addition, poor sealing of cremation equipment enables odor to escapes to the exterior environment through doors and windows and is the main source of onsite odor. Large variations in factors such as the intensity of cremation on the day of monitoring, the monitoring time and the degree of ventilation in the workshop produce wide fluctuations in the odor concentration in the cremation workshop.

### Correlation analysis

**Fig. 6:**
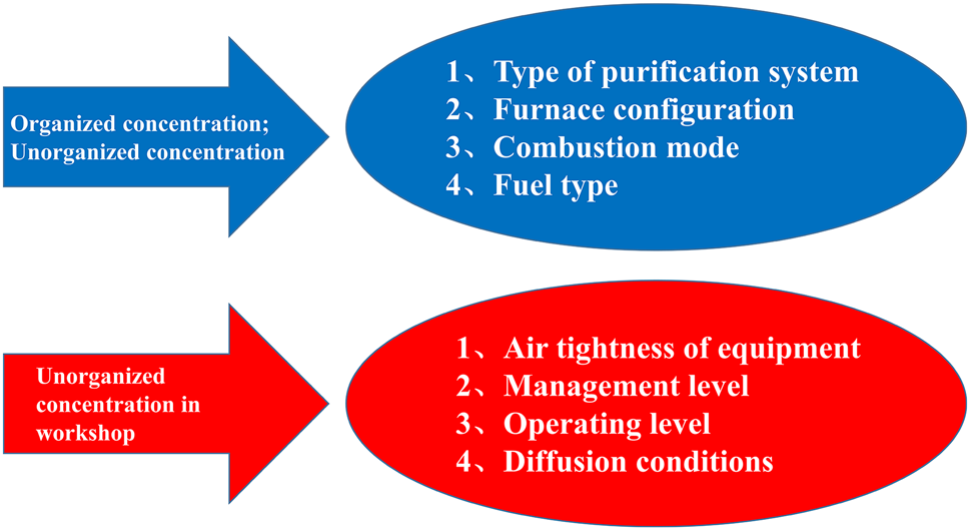
Correlation analysis

We analyzed the level and influencing factors of the organized and unorganized odor emissions from the Beijing crematoriums. We found that the flue gas purification system, furnace structure, combustion method, and fuel type strongly impact the organized emissions from cremators and incinerators. Current postprocessing facilities installed on the equipment have no effect on reducing the odor concentration, resulting in a high level of organized odor emissions, which affects the level of unorganized odor emissions within the crematorium boundaries but has little effect on the level of unorganized odor emissions within the workshop, which is mainly associated with the airtightness of the equipment, operation management, operation skill of the personnel, and workshop ventilation conditions (Fig. 6). Although crematoriums have been upgraded and personnel operational skills have improved, the odor concentration in workshops has not been effectively controlled and affects the concentration of unorganized odor emissions within the crematorium boundaries through diffusion, which is currently the focus of odor control in crematoriums.

### Comparison of odor levels of crematoriums and other industries

Currently, the organized odor emission level of crematoriums in Beijing meets the regulatory standards because the Emission Standard of Air Pollutants for Crematoriums (GB 13801-2015) does not include an odor concentration limit. In addition, the current upper limit on the emission concentration set by the Beijing Municipal Emission Standard of Air Pollutants for Crematoriums (DB11/1203-2015) is rather high and mainly intended for the regulation of emissions of waste gases from the production process and other atmospheric pollutants discharged from industrial furnaces and kilns. Compared with waste discharge units, such as those of industrial furnaces and kilns, crematoriums have a lower operating intensity and do not use raw and auxiliary materials except for fuels, thereby generating a lower level of odor. To further analyze the odor concentration level of crematoriums and prove the necessity of odor control, reported odor concentrations for common industrial furnaces and kiln industries were compared with those for crematoriums.

**Fig. 7:**
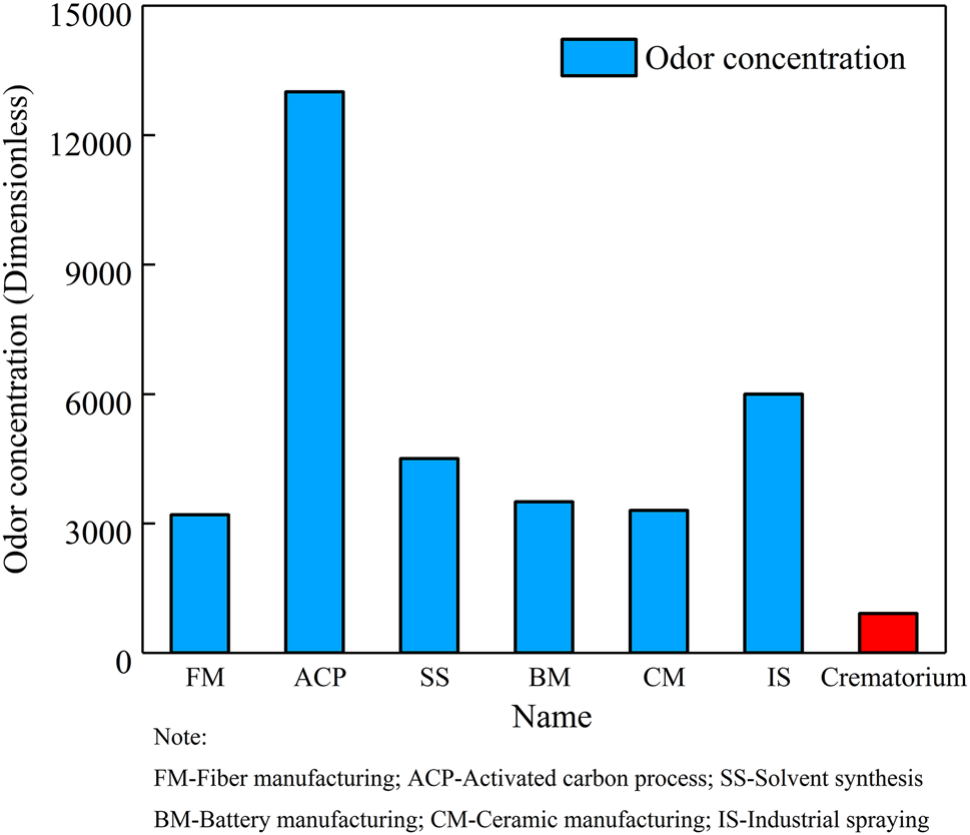
Comparison with other industries' odor levels

Meng et al. the characterized the odor pollution emissions from industrial parks and determined the odor concentrations for fiber manufacturing, activated carbon processing, solvent synthesis, battery manufacturing, ceramic manufacturing, and coating industries [14]: the odor concentration of crematoriums is only 2/7, 1/14, 1/5, 1/4, 2/7 and 2/13 of those of the aforementioned industries, respectively (Fig. 7). However, as crematoriums have unique locations, the generated odor has a higher impact on the surrounding residents than industries, such that emission pollution from crematoriums cannot be neglected. It is recommended that the limits of organized and unorganized odor emissions in industry standards be further clarified and reduced to prompt crematoriums to improve odor control in workshops, while strengthening deodorization measures and comprehensively managing emissions.

### Emission reduction potential and control measures

An analysis of the data and the field investigation results obtained in this study showed that some crematoriums in Beijing have not yet met the standards on odor emissions, especially regarding unorganized odor emissions for workshops. Crematoriums can reduce odor emissions mainly through the comprehensive management and control of unorganized emissions in workshops, improving the effectiveness of waste gas treatment, and the installation of deodorizing devices. Based on these considerations, we propose the following control measures.

1. For comprehensive management and control of unorganized odor emissions in workshops, workshop ventilation should be improved, and exhaust fans should be installed considering practical conditions, such that low-concentration unorganized odor emissions can be promptly diluted and discharged. Additionally, equipment should be operated in an intermittent working mode to reduce odor accumulation in the workshop associated with the workload. Unified technical specifications for crematorium operations should be formulated to better standardize operation, thereby preventing unorganized odor emissions produced by improper operations. Equipment should be regularly inspected to ensure proper functioning well and sufficiently airtightness to mitigate the risk from crematorium odor emissions to the staff and the surrounding environment.
2. Currently, cremators and incinerators are generally not equipped with odor-reducing devices, and odor emissions are mainly lowered through coordinated reduction. Commonly used methods for lowering odor emission levels include dilution and electroadsorption [15–18]. Dilution achieves odor reduction by passing the flue gas through a secondary combustion chamber, in which the odor generated in the main combustion chamber is burned again at a high temperature to generate CO, carbon dioxide, NOx, etc., while fresh air is blown through to dilute the odor [19–21]. Electroadsorption is a novel technique for purifying the odor in flue gas and removing odorous gas [22–25], albeit at a typically high cost. In recent years, the rapid development of biotechnology and low-temperature plasma technology has provided novel promising measures for controlling low-concentration odors. These technologies could be gradually applied for odor control in crematoriums.
3. It is very important to revise emission standards to enable policy control of odor emissions. Currently, there is no limit on odor emissions in the Beijing Municipal Emission Standard of Air Pollutants for Crematoriums (DB11/1203-2015), and the odor concentration limit remains at the high value specified in the Emission Standard of Air Pollutants for Crematoriums (GB 13801-2015). To further mitigate odor emissions from crematoriums in Beijing, it is recommended that the odor emission limit be taken into account in evaluating the implementation effectiveness of the industry standard. Enterprises will be thus be prompted to adopt corresponding control measures and construct deodorizing devices as soon as possible, ensuring that odors emitted during the operation of crematoriums are efficiently trapped and controlled.

## Conclusion

In general, the crematoriums in Beijing are clean, and the installation of postprocessing facilities has effectively controlled emissions of most pollutants from cremators and incinerators. However, the problem of odor emissions has not yet been effectively resolved and continues to impact the surrounding environment and residents. In this study, we used monitoring data collected from five crematoriums in Beijing to investigate the level and influencing factors of odor emissions. We compared the results with those of other industries and proposed corresponding emission reduction potential and control measures.

First, the concentration of unorganized odor emissions from the cremation workshops in Beijing was found to be rather high, ranging from 97 to 732 (504 on average), which can impact the health of the workshop operators and the surrounding environment. This high concentration mainly results from poor ventilation conditions in cremation workshops that facilitate the accumulation of odor during the cremation process. Additionally, the aging and poor sealing of cremation equipment leads to an increase in the odor concentration in cremation workshops. Odor escapes from the equipment through doors and windows, becoming the main source of onsite odor. Variations in factors such as the intensity of cremation, monitoring time and the degree of ventilation in the workshop strongly impact the monitored range of odor concentration in cremation workshops.

Second, the measured concentrations of organized odor emissions in cremators and incinerators ranged from 231 to 1303 (910 on average). These values meet current implementation standards, though these standards are mandated for industries with industrial furnaces and kilns and are not applicable to crematoriums. The odor emission level of crematoriums was found to be lower than those of other industries, including industrial furnaces. Nevertheless, it is necessary to strengthen the management and control of odor emissions from crematoriums because the unique geographical locations of crematoriums and high population density of the surrounding areas creates a high exposure risk.

Third, there is still room for improvement of the level of odor emissions from crematoriums in Beijing. The current implemented upper limit of odor emission concentration is rather high. To further mitigate odor emissions in Beijing, it is recommended that industry standards be clarified and the upper limit of organized and unorganized odor emissions be lowered. Crematoriums will thus be prompted to strengthen odor trapping measures by adopting various methods, such as installing exhaust fans, and applying control measures, such as adsorption and biological treatment, to control odor pollution at the source.
